# Health economic analysis of third-line interventions in diffuse large B-cell lymphomas in Germany: applying the efficiency frontier

**DOI:** 10.1186/s12962-022-00400-0

**Published:** 2022-12-12

**Authors:** Florian Jakobs, Julia Jeck, Paymon Ahmadi, Anna Kron, Florian Kron

**Affiliations:** 1 Department of Hematology and Stem Cell Transplantation, University Hospital Essen, University of Duisburg-Essen, Essen, Germany; 2 VITIS Healthcare Group, Cologne, Germany; 3 Faculty of Medicine and University Hospital Hamburg-Eppendorf, Center for Oncology, University of Hamburg, Hamburg, Germany; 4 Department I of Internal Medicine, Faculty of Medicine and University Hospital Cologne, University of Cologne, Cologne, Germany; 5 National Network Genomic Medicine Lung Cancer, University Hospital Cologne, Cologne, Germany; 6 Faculty of Medicine and University Hospital Cologne, Center for Integrated Oncology (CIO ABCD), University of Cologne, Cologne, Germany; 7 FOM University of Applied Sciences, Essen, Germany

**Keywords:** DLBCL, Efficiency frontier, CAR T-cell therapy, Stem cell transplantation, Best supportive care

## Abstract

**Background:**

In the past decades, highly innovative treatments in the field of diffuse large B-cell lymphoma (DLBCL) became available in clinical practice. The aim of this study was to assess the cost–benefit relation of third-line interventions in DLBCL from a German payer perspective.

**Methods:**

Clinical benefit of allogeneic stem cell transplantation (alloSCT), chimeric antigen receptor T cells therapy (CAR T) [tisagenlecleucel (tisa-cel) and axicabtagene ciloleucel (axi-cel)] and best supportive care (BSC) was assessed in terms of median overall survival (median OS) derived from a systematic literature review in PubMed. Real-world treatment costs were retrieved from the university hospitals Cologne and Hamburg-Eppendorf. The cost–benefit relation was analysed using the efficiency frontier concept.

**Results:**

Median OS varied from 6.3 months in BSC to 23.5 months in CAR T (axi-cel), while median real-world treatment costs ranged likewise widely from €26,918 in BSC to €340,458 in CAR T (axi-cel). Shown by the efficiency frontier, alloSCT and axi-cel were found as most efficient interventions.

**Conclusion:**

The efficiency frontier supports the pricing of innovative therapies, such as third-line interventions in DLBCL, in relation to appropriate comparators. Yet, studies with longer follow-up periods are needed to include studies with unreached median OS and to reflect experiences gained with CAR T in clinical practice.

## Introduction

Diffuse large B-cell lymphoma (DLBCL) is the most common malignant sub-type of the non-Hodgkin-lymphoma, reaching an incidence rate of 12.3 per 100,000 inhabitants in Germany in 2017 [1]. Moreover, DLBCL as an aggressive lymphoma progresses rapidly while spreading lymphoma cells to the organism in early stages of the disease already. Thereby, age (> 60 years) and Eastern Cooperative Oncology Group performance status (ECOG PS ≥ 2) are two exemplary prognostic risk factors promoting the refractoriness/relapse of DLBCL within the international prognostic index (IPI) [2, 3]. While patients with an early initiation of a first-line treatment face a 10-year overall survival (OS) rate of 43.5%, treatment prognosis deteriorates when patients receive no therapy, or relapse or become refractory to prior treatment line [4–6].

An enormous amount of research has been conducted in the field of DLBCL in the past decades, leading to highly innovative and promising treatment approaches for clinical practice. For instance allogeneic hematopoietic stem cell transplantation (alloSCT) and chimeric antigen receptor T cells therapy (CAR T) has shown promising clinical improvement for patients with progressive disease who had received two or more lines of systemic therapy before [7, 8] or are refractory or relapse after high-dose chemotherapy and autologous stem cell transplantation [9]. Such innovative treatment options for small patient populations (orphan drugs) often incur higher costs from a payers’ perspective due to highly individualized therapies [10]. Thus, comparative economic evaluations regarding efficiency are challenging and different health economic methods are required for the price determination of innovative treatments.

Previous international studies investigated and modelled cost-effectiveness of DLBCL third-line interventions [11–14]. As to our knowledge a health economic evaluation in the German healthcare context is currently lacking. Thus, the aim of this study was to analyse third-line interventions of DLBCL regarding their efficiency and costs in Germany.

## Methods

We compared the costs and benefits of third-line interventions in DLBCL by applying the efficiency frontier as health economic evaluation method. This approach is recommended by the German Institute for Quality and Efficiency in Health Care (IQWiG) and intends to assess the relation of incurred treatment costs and clinical benefit for different interventions within one indication [15]. Displayed in a two-dimensional graph, with costs on the x-axis and the benefit on the y-axis, the most efficient interventions result in the efficiency frontier, which is increasingly used and discussed in health economics [16–18].

Based on the respective labels of the DLBCL third-line interventions approved by the European Medicines Agency (EMA), we identified the treatment options on 2021-03-05: alloSCT, two CAR T treatments (tisagenlecleucel (tisa-cel) [7] and axicabtagene ciloleucel (axi-cel) [8]). We further assumed best supportive care (BSC) including palliative procedures as alternative intervention. [19]

### Determination of clinical benefits

Clinical benefit was measured as median overall survival (median OS) as a primary patient-relevant outcome (PRO). We followed the official IQWiG guidelines for searching publications in bibliographic databases [15] and the PRISMA statement [20] to conduct a systematic literature review in PubMed (MEDLINE). The search terms *CAR T cell therapy*, *allogeneic stem cell transplantation* and *best supportive care* were respectively combined with the search term *diffuse large B-cell lymphoma* by the Boolean Operator “AND”. Combinations of treatment options and indication were added by the operator “OR”. For each of the search terms, synonyms, similar concepts and different spellings were defined and inserted by the operator “OR”. If applicable, medical subject headings (MeSH terms) were used. Two researchers conducted the systematic literature review individually to avoid biases. Discrepancies were resolved by discussion.

Articles were included if the indication was DLBCL in a third-line setting with EMA approval and guideline recommendation of the European Society for Medical Oncology [7–9], the median OS was reported, and the article was written in English language. Articles were excluded if the study population was ≤ 18 years, the study was not based on human beings or articles were published prior to 2017. Literature reviews, case studies, and case series with less than 15 patients were also not considered. Furthermore, articles were not considered if the median OS was not reached during the studies’ assessment period or median OS was not distinguished by different CAR T products. If multiple median OS values for the same third-line intervention were found, their median was calculated by meta-analysis for the efficiency frontier.

### Determination of treatment costs

To quantify treatment costs from a healthcare payers’ perspective, we analysed real-world data of each third-line intervention which patients incurred during the entire hospital stay of cell administration or palliative medical care. Costs were retrieved from the data warehouses of the University Hospital Cologne and the University Hospital Hamburg-Eppendorf. Thereby, we initially searched for the main diagnosis diffuse large B-cell lymphoma, specified by the International Statistical Classification of Diseases code (ICD-10-GM version 2021) C83.3. In a next step, third-line therapies were identified by the operating and procedure (OPS) codes shown in Table 1. As the CAR T OPS codes do not differentiate between commercial CAR T products, patient files were screened for further clarification. Treatment costs were analyzed in terms of German Diagnosis Related Group (G-DRG) tariffs to reflect the payers’ perspective. Besides G-DRG tariffs, additional fees for new treatment methods (NUB, “Neue Untersuchungs- und Behandlungsmethoden”; ZE, “Zusatzentgelte”) were considered. Case characteristics, median treatment costs and ranges were calculated for each third-line intervention, while the treatment option CAR T was further differentiated by the products tisa-cel and axi-cel. If a patient with cell therapy received palliative medical care at a later point during the hospital stay, the patient was assigned to the third-line intervention in which higher costs were caused.

**Table 1:** Operating and procedure codes for the identification of third-line interventions

Third-line intervention	OPS Code	Description
CAR T	8-802.24	Transfusion of leukocytes (1–5 TU) with genetically and tumour-specific in vitro preparation including CAR T-cells
	8-802.34	Transfusion of leukocytes (more than 5 TU) with genetically and tumour-specific in vitro preparation including CAR T-cells
alloSCT	8-805.2	Transfusion of peripheral hematopoietic stem cells, allogeneic, not HLA-identical, related donor
	8-805.3	Transfusion of peripheral hematopoietic stem cells, allogeneic, not HLA-identical, unrelated donor
	8-805.4	Transfusion of peripheral hematopoietic stem cells, allogeneic, HLA-identical, related donor
	8-805.5	Transfusion of peripheral hematopoietic stem cells, allogeneic, HLA-identical, unrelated donor
BSC	1-774	Standardized palliative medical basic assessment
	8-982	Palliative medical complex treatment
	8-98e	Specialized inpatient palliative medical complex treatment
	8-98h	Specialized palliative medical complex treatment by a palliative service

HLA, human leukocyte antigens; TU, transfusion unit

Due to the 1-year study period (discharge date from 01-06-2019 to 01-06-2020) and in accordance with the German recommendation on health economic evaluation and the General Methods of the IQWiG, no discounting of the treatment costs was applied [15, 21]. Economic values were given in Euro (€).

## Results

### Clinical benefits

**Fig. 1:**
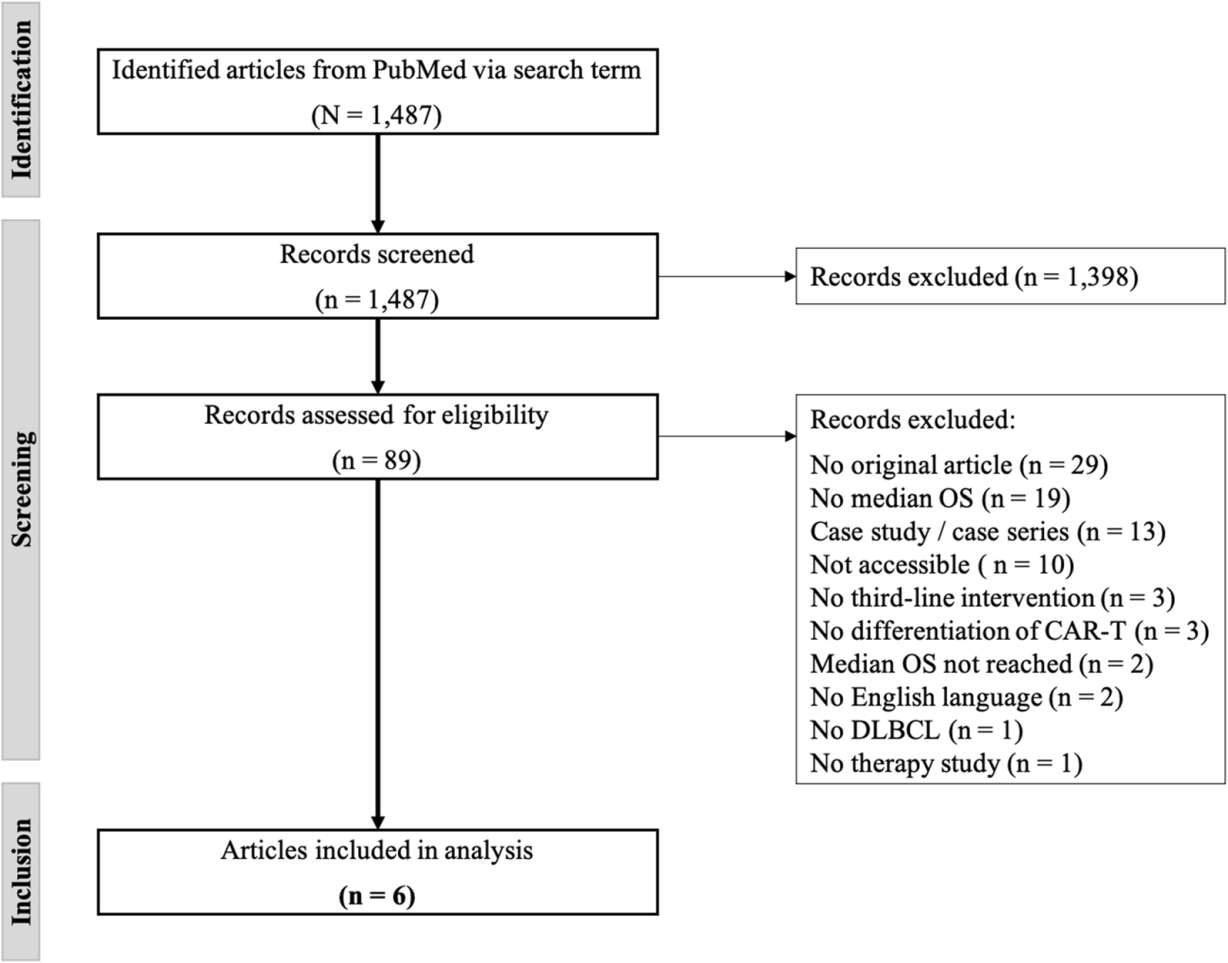
PRISMA flow diagram

In total, 1487 records were identified in PubMed via search term on the reporting date 2021-03-05 (Fig. 1). By screening of titles and abstracts considering the predefined exclusion criteria, 1398 records were removed, mainly due to the publication date prior 2017, secondary data, deviating treatment regimens, or as they were case series with less than 15 cases. Thus, 89 records were further assessed for eligibility by analysing full texts, leading to an exclusion of another 83 records. The most common reasons for exclusion were: No original article (n = 29), no indication of median OS (n = 19) and a small study population in case studies or series (n = 13). The systematic literature review resulted in six articles which were included for the analysis of clinical benefits, summarized in Table 2.

**Table 2:** Results of the systematic literature review regarding clinical benefits

Authors	Title	Year	Journal	Intervention	Median OS
Crump et al. [22]	Outcomes in refractory diffuse large B-cell lymphoma: results from the international SCHOLAR-1 study	2017	Blood	BSC	6.3 months
Dreger et al. [23]	CAR T cells or allogeneic transplantation as standard of care for advanced large B-cell lymphoma: an intent-to-treat comparison	2020	Blood Advances	alloSCT	7.4 months^a^
Sesques et al. [24]	Commercial anti-CD19 CAR T cell therapy for patients with relapsed/refractory aggressive B cell lymphoma in a European center	2020	American Journal of Hematology	CAR T	11.8 months (tisa-cel) not reached (axi-cel)
Dean et al. [26]	High metabolic tumor volume is associated with decreased efficacy of axicabtagene ciloleucel in large B-cell lymphoma	2020	Blood Advances	CAR T	34 months (axi-cel)
Schuster et al. [25]	Tisagenlecleucel in Adult Relapsed or Refractory Diffuse Large B-Cell Lymphoma	2019	New England Journal of Medicine	CAR T	12 months (tisa-cel)
Mian et al. [27]	Outcomes and factors impacting use of axicabtagene ciloleucel in patients with relapsed or refractory large B-cell lymphoma: results from an intention-to-treat analysis	2020	Leukemia & Lymphoma	CAR T	13 months (axi-cel)

^a^Study reported median OS of 222 days (assumption: 30 days = 1 month)

**Fig. 2:**
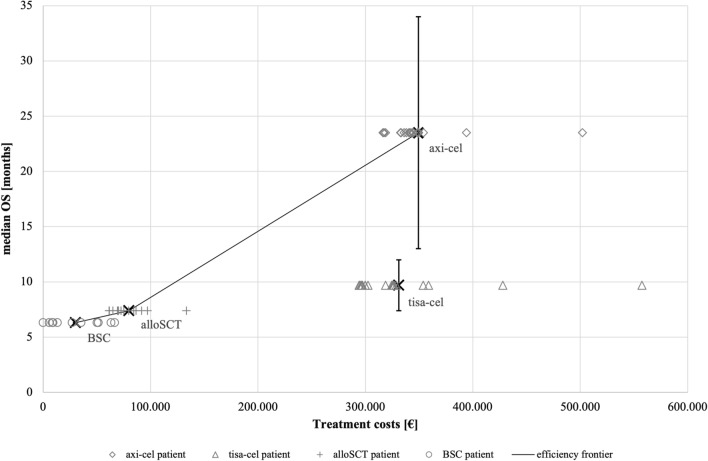
Efficiency frontier in third-line therapies in DLBCL

Median OS for BSC and alloSCT was reported with 6.3 months [22] and 7.4 months [23], respectively. Considering CAR T, two articles for each product fulfilled the inclusion criteria and thus, the median was calculated. The resulting median OS values were 9.7 months for tisa-cel [24, 25] and 23.5 months for axi-cel [26, 27]. The range of median OS values was indicated by antennas in Fig. 2.

### Treatment costs

In both University Hospitals, a total of 62 patients (N = 62 cases) receiving third-line intervention in DLBCL were identified (Table 3). The population size per intervention varied with 17.7% (n = 11) in BSC, 22.58% (n = 14) in alloSCT, 27.42% (n = 17) in axi-cel and 32.26% (n = 20) in tisa-cel. Percentage shares based on sex were mostly balanced. However, a difference in age was found. While the median age of BSC, tisa-cel and axi-cel were similar with 63, 60 and 62 years, respectively, alloSCT patients had a median age of 49, reaching a maximum of 66 years.

**Table 3:** Case characteristics and treatment costs by third-line interventions

	BSC	alloSCT	CAR T	
			tisa-cel	axi-cel
Patients no. (%)	11 (17.74)	14 (22.58)	20 (32.26)	17 (27.42)
*Sex no. (%)*				
Female	3 (4.84)	7 (11.29)	12 (19.35)	6 (9.68)
Male	8 (12.90)	7 (11.29)	8 (12.90)	11 (17.74)
Age^a^	63 [32–83]	49 [20–66]	60 [23–82]	62 [24–79]
Casemix index†	6.97 [0.98–31.25]	14.38 [10.56–16.98]	1.70 [1.18–47.71]	3.17 [1.59–29.52]
Length of stay in days^a^	52 [13–130]	38 [23–51]	21 [12–110]	28 [19–111]
Number of side diagnoses^a^	18 [5–47]	19 [13–41]	11.5 [2–51]	21 [10–43]
Treatment costs^a^	€ 26,918 [€ 0.00–€ 66,468]	€ 73,829 [€ 61,337–€ 133,280]	€ 310,496 [€ 294,113–€ 557,423]	€ 340,458 [€ 316,272–€ 502,096]

^a^Median values [range]

The casemix index (CMI), indicating the cases’ resource intensity, ranged from 1.70 in tisa-cel to 14.38 in alloSCT. Considering CAR T products, CMI, length of stay (LOS) and number of side diagnoses were lower in tisa-cel patients. Even though the median LOS was clearly the longest in BSC with 52 days, the maximum LOS of both CAR T products and BSC were comparably long. Median treatment costs (G-DRG tariffs and potentially additional fees) across all cases differed, ranging from € 26,918 in BSC to € 340,458 in axi-cel. The ranges of treatment costs for both CAR T products were largely overlapping. The case-based treatment costs are also displayed in Fig. 2 clustered per intervention.

### Efficiency frontier

Combining the results of clinical benefits as measured in median OS and treatment costs based on real-world data, the efficiency frontier for the third-line treatment interventions in DLBCL was plotted (Fig. 2). The treatment cost distribution of each intervention was depicted by individual data points. The range of values underlying in the median OS for axi-cel and tisa-cel were additionally displayed as whiskers. The graph showed that alloSCT and axi-cel form the efficiency frontier at increasing levels of median OS. Further, BSC is an efficient alternative when medically required. Interventions that are below the efficiency frontier are less desirable as they generate less or equal benefits (herein: median OS) at higher costs than other existing interventions. The CAR T product tisa-cel with 9.7 months median OS was of a greater medical benefit for patients compared to BSC and alloSCT. Based on the underlying efficiency frontier, tisa-cel is less efficient compared to axi-cel based on its cost–benefit relation.

## Discussion

The medical benefit of the DLBCL third-line interventions has already been extensively investigated in previous studies [28]. However a health economic evaluation in the German healthcare context is currently lacking. To our knowledge, this study was the first health economic analysis putting treatment costs and median OS of the underlying interventions into relation. With the efficiency frontier, we analyzed the currently approved third-line interventions in DLBCL from a health economic perspective. With regard to the two assessed CAR T products, we showed that axi-cel was more efficient than tisa-cel when comparing the ratio of real-life treatment costs and median OS as clinical benefit.

As part of the treatment costs analysis, attention should be drawn to a particularity of the G-DRG system: The CMI for inpatient treatments reflects the complexity of a treatment and thus mostly treatment costs. In the case of the treatment with both CAR T products, tisa-cel and axi-cel, the low CMI in relation to the high treatment costs can be explained by the fact that the CMI only reflects diagnosis and OPS codes considered in the tariffs. Due to the adaptative nature of the G-DRG system, further cost data need to be acquired and evaluated to adequately consider CAR T in DRG tariffs. The manufacturing and administration process of CAR T, however, is currently remunerated by locally negotiated additional fees at the hospital level. Being reimbursed with around €265,000 (tisagenlecleucel) and €282,000 (axicabtagene ciloleucel) in 2022 [29, 30], this still makes up a large part of the overall treatment costs. The exemplary reimbursement amounts were retrieved from publicly available tariffs of two university hospitals, as it is not obligated to publish negotiation results.

The intervention of BSC differs from the other two interventions as not the disease itself is combated but accompanied symptoms or side effects shall be mitigated by a variety of patient-specific medical measures, often leading to a longer LOS. The wide LOS ranges in CAR T products were attributable to patients who received palliative medical care after cell administration.

### Methodological reflection and further research

Even though the analysis was carried out in accordance with the highest health economic standards, differences in the underlying study population, previous therapies or allowance regarding bridging therapy have not been taken into account. In particular, the similarity of existing studies comparing CAR T treatments are currently focused by scientific discourse [31–34]. Using the indirect treatment comparison, Zhang et al. assessed the data from the respective pivotal studies ZUMA-1 [35] and JULIET [25] to be not comparable [31]. The comparability attested by Oluwole et al. [32] using a matching-adjusted indirect comparative (MAIC) analysis was again questioned by a letter to the editor [33]. However, Oluwole et al. considered MAIC as well-established methodological approach to perform cross-trial comparison while having transparently outlined limitations of their study [34].

The herein conducted systemic literature review also builds the standard in research to identify, select and critically appraise available knowledge. Considering third-line interventions in patients with DLBCL, the availability of scientific articles reporting a median OS, is very limited for innovative treatments such as CAR T. Potentially leading to a time-lag bias, the review only considered articles as of 2017 to make results of different interventions more comparable. The median OS, however, may be imprecise as an indicator for the long-term survival of patients e.g. if the mortality rate is particularly high shortly after the start of therapy and flattens out in the further course of observation. To further increase the comparability and topicality in determining the median OS in this systematic literature review, only studies after the introduction of CAR T were considered. Real-life cost data of the third-line interventions were also retrieved from a timepoint when the reimbursement of CAR T was already firmly established. Several studies had to be excluded as the median OS has not been reached. Thus, comparative studies with longer follow-ups or updated Kaplan–Meier curves are indispensable to consider studies with currently unreached median OS. This would help to better reflect experience gained in clinical practice while potentially giving a better overview on potentially improved survival or a decrease in side effects. This in turn, may have an impact on resource consumption. In addition, an evaluation of the entire patient journey and across sector boundaries would enable a holistic cost analysis of the therapies and follow-up appointments of DLBCL patients.

## Conclusion

In the German healthcare context, the Act on the Reform of the Market for Medicinal Products (“AMNOG—Nutzenbewertung von Arzneimitteln, § 35a SGB V”) builds the legal obligation for the benefit assessment of new pharmaceutical in comparison to the appropriate comparative therapy. In case of third-line interventions in DLBCL, no appropriate comparator for CAR T were approved as both commercial CAR T products were granted almost simultaneously. The underlying efficiency frontier, however, can provide support for decision makers by indicating cost-covering and reimbursement in relation to the best currently available third-line intervention in DLBCL. For this purpose, it is also used by the Belgian Healthcare Knowledge Centre (KCE) and the Haute Autorité de Santé (HAS) in France [36, 37]. Without considering multiple PROs (i.e. the occurrence of adverse events or comorbidities) at the same time, the efficiency frontier is only one important part in the comprehensive evaluation and price determination of innovative therapies. Thus, recurring health economic evaluations are needed to master the increasing cost pressure especially in health care systems that are largely financed by social insurance contributions, likewise in Germany.

## Appendices

### Appendix 1: Search term

(("lymphoma, large b cell, diffuse/diagnosis"[MeSH Terms] OR "DLBCL"[Title/Abstract] OR "rDLBCL"[Title/Abstract] OR "refractory dlbcl"[Title/Abstract] OR "refractory diffuse large b cell lymphoma"[Title/Abstract] OR "relapsed dlbcl"[Title/Abstract] OR "recurrent dlbcl"[Title/Abstract] OR "large b-cell lymphoma"[Title/Abstract] OR "large b-cell lymphoma"[Title/Abstract]) AND ("antigens, cd19"[MeSH Terms] OR "cell and tissue based therapy"[MeSH Terms] OR "receptors, antigen, t cell"[MeSH Terms] OR "anti cd19 car t cell therapy"[Title/Abstract] OR "car t therapy"[Title/Abstract] OR "car t cell therapy"[Title/Abstract] OR "car t cell therapy"[Title/Abstract] OR "car t"[Title/Abstract] OR "chimeric antigen receptor"[Title/Abstract] OR "Tisagenlecleucel"[Title/Abstract] OR "Kymriah"[Title/Abstract] OR "axicabtagene ciloleucel"[Title/Abstract] OR "Yescarta"[Title/Abstract] OR "axicabtagene ciloleucel"[Title/Abstract] OR "axi-cel"[Title/Abstract] OR "tisa-cel"[Title/Abstract])) OR (("lymphoma, large b cell, diffuse/diagnosis"[MeSH Terms] OR "DLBCL"[Title/Abstract] OR "rDLBCL"[Title/Abstract] OR "refractory dlbcl"[Title/Abstract] OR "refractory diffuse large b cell lymphoma"[Title/Abstract] OR "relapsed dlbcl"[Title/Abstract] OR "recurrent dlbcl"[Title/Abstract] OR "large b-cell lymphoma"[Title/Abstract] OR "large b-cell lymphoma"[Title/Abstract]) AND ("adult stem cells"[MeSH Terms] OR "allografts"[MeSH Terms] OR "transplantation, homologous"[MeSH Terms] OR "hematopoietic stem cell transplantation"[Title/Abstract] OR "HSCT"[Title/Abstract] OR "allo-HSCT"[Title/Abstract] OR "allo-HSCT"[Title/Abstract])) OR (("lymphoma, large b cell, diffuse/diagnosis"[MeSH Terms] OR "DLBCL"[Title/Abstract] OR "rDLBCL"[Title/Abstract] OR "refractory dlbcl"[Title/Abstract] OR "refractory diffuse large b cell lymphoma"[Title/Abstract] OR "relapsed dlbcl"[Title/Abstract] OR "recurrent dlbcl"[Title/Abstract] OR "large b-cell lymphoma"[Title/Abstract] OR "large b-cell lymphoma"[Title/Abstract]) AND ("palliative care"[MeSH Terms] OR "best supportive care"[Title/Abstract] OR "BSC"[Title/Abstract] OR "salvage therapy"[Title/Abstract] OR "adjuvant therapy"[Title/Abstract] OR "Palliation"[Title/Abstract] OR "palliative treatment"[Title/Abstract] OR "palliative chemotherapy"[Title/Abstract] OR "chemotherapy regimen"[Title/Abstract])).

### Appendix 2: Treatment costs per case

**Table:** 

Case ID	Discharge date	G-DRG tariff	Length of stay in days	Third-line intervention	CAR T product	Treatment costs in EUR		
						G-DRG tariff	Additional fee	Total
1	03.07.2019	R61D	30	CAR T	Yescarta	9759.88	331,537.58	341,297.46
2	18.07.2019	R61A	42	CAR T	Yescarta	17,798.27	336,083.92	353,882.19
3	15.07.2019	R61E	21	CAR T	Yescarta	6147.21	327,000.00	333,147.21
4	16.07.2019	R61B	19	CAR T	Yescarta	11,202.13	327,000.00	338,202.13
5	09.08.2019	R61A	23	CAR T	Yescarta	17,116.03	331,863.53	348,979.56
6	15.08.2019	R61E	20	CAR T	Yescarta	6147.21	327,000.00	333,147.21
7	17.08.2019	R61B	22	CAR T	Yescarta	11,202.13	329,255.74	340,457.87
8	06.09.2019	A04E	38	AlloSCT		50,842.61	40,716.21	91,558.82
9	31.08.2019	R61A	32	CAR T	Yescarta	17,116.03	327,000.00	344,116.03
10	22.08.2019	R61E	20	CAR T	Yescarta	5609.90	327,000.00	332,609.90
11	10.09.2019	A04E	31	AlloSCT		50,842.61	46,234.69	97,077.30
12	27.09.2019	A04D	40	AlloSCT		57,516.52	17,665.40	75,181.92
13	25.09.2019	A04D	23	AlloSCT		57,516.52	7426.86	64,943.38
14	04.10.2019	R61H	22	CAR T	Yescarta	7826.35	328,116.97	335,943.32
15	28.10.2019	A04D	40	AlloSCT		57,516.52	11,556.85	69,073.37
16	22.10.2019	R61B	29	CAR T	Yescarta	11,202.13	333,469.57	344,671.70
17	07.11.2019	R61B	34	CAR T	Yescarta	11,817.20	330,525.85	342,343.05
18	27.11.2019	A36A	37	AlloSCT		60,019.24	73,261.02	133,280.26
19	15.11.2019	R61F	13	BSC		5143.29	600.00	5743.29
20	24.12.2019	A04E	32	AlloSCT		50,842.61	33,567.03	84,409.64
21	18.03.2020	A15C	111	CAR T	Yescarta	104,353.71	397,742.01	502,095.72
22	05.02.2020	A04D	51	AlloSCT		57,516.52	21,996.62	79,513.14
23	14.01.2020	R61B	28	CAR T	Yescarta	11,202.13	307,573.49	318,775.62
24	24.02.2020	R61A	12	CAR T	Kymriah	13,213.10	305,478.67	318,691.77
25	22.03.2020	A04E	27	AlloSCT		38,729.83	31,039.44	69,769.27
26	05.05.2020	R61A	62	CAR T	Kymriah	24,302.78	329,544.72	353,847.50
27	23.04.2020	A04D	45	AlloSCT		43,706.29	26,440.86	70,147.15
28	21.05.2020	A11B	64	CAR T	Yescarta	62,684.34	331,238.04	393,922.38
29	24.04.2020	A04E	24	AlloSCT		38,729.83	22,846.04	61,575.87
30	02.07.2020	R61B	27	CAR T	Yescarta	8585.03	308,711.92	317,296.95
31	26.09.2020	A36B	107	CAR T	Kymriah	74,499.99	353,269.58	427,769.57
32	30.07.2020	R03Z	38	CAR T	Yescarta	11,918.56	304,353.43	316,271.99
33	17.02.2020	R07B	18	BSC		6042.69	2595.93	8638.62
34	09.06.2020	R61G	26	BSC		9245.40	4033.23	13,278.63
35	07.10.2019	R61A	52	BSC		24,635.19	26,791.43	51,426.62
36	24.06.2020	A04E	50	AlloSCT		38,700.26	22,636.80	61,337.06
37	16.12.2019	A04E	40	AlloSCT		50,872.67	35,453.61	86,326.28
38	04.12.2019	A04E	34	AlloSCT		50,872.67	21,603.91	72,476.58
39	09.06.2020	R61H	21	CAR T	Kymriah	5082.58	292,104.86	297,187.44
40	05.05.2020	R11A	21	CAR T	Kymriah	8028.81	291,729.11	299,757.92
41	08.10.2019	R61H	20	CAR T	Kymriah	5280.73	320,000.00	325,280.73
42	29.06.2020	R61H	20	CAR T	Kymriah	4793.09	291,882.20	296,675.29
43	05.06.2020	R61B	24	CAR T	Kymriah	8578.48	293,720.69	302,299.17
44	27.01.2020	R61H	20	CAR T	Kymriah	4793.09	291,029.40	295,822.49
45	23.01.2020	R61H	20	CAR T	Kymriah	4793.09	291,029.40	295,822.49
46	31.01.2020	R61A	59	CAR T	Kymriah	29,413.67	294,963.38	324,377.05
47	18.11.2019	R61H	20	CAR T	Kymriah	6009.35	288,500.00	294,509.35
48	18.09.2019	R61H	15	CAR T	Kymriah	4187.80	324,357.69	328,545.49
49	16.09.2019	R61H	20	CAR T	Kymriah	6009.35	320,000.00	326,009.35
50	04.12.2019	R61E	22	CAR T	Kymriah	5613.22	288,500.00	294,113.22
51	03.09.2019	R61H	13	BSC		3459.18	5749.45	9208.63
52	02.08.2020	R03Z	77	BSC		29,058.92	21,171.77	50,230.69
53	05.02.2020	A11A	110	CAR T	Kymriah	168,760.88	388,662.45	557,423.33
54	18.09.2019	R01A	63	BSC		34,425.60	769.08	35,194.68
55	01.04.2020	A15C	58	BSC		42,067.81	21,247.74	63,315.55
56	05.03.2020	R61D	21	CAR T	Kymriah	5558.97	291,459.95	297,018.92
57	05.12.2019	R61B	23	CAR T	Kymriah	11,208.75	288,500.00	299,708.75
58	09.01.2020	R61B	37	BSC		13,670.51	13,247.76	26,918.27
59	21.08.2019	R61E	20	CAR T	Kymriah	5613.22	321,200.50	326,813.72
60	27.10.2020		130	BSC		0.00	0.00	0.00
61	30.03.2020	R16Z	82	BSC		40,349.15	26,118.84	66,467.99
62	15.01.2020	A36B	51	CAR T	Kymriah	42,107.99	316,610.64	358,718.63

### Appendix 3: PRISMA checklist*

**Table:** 

Section/topic	#	Checklist item	Reported on page #
*1. Title*			
Title	1	Identify the report as a systematic review, meta-analysis, or both	n/a
*2. Abstract*			
Structured summary	2	Provide a structured summary including, as applicable: background; objectives; data sources; study eligibility criteria, participants, and interventions; study appraisal and synthesis methods; results; limitations; conclusions and implications of key findings; systematic review registration number	n/a
*3. Introduction*			
Rationale	3	Describe the rationale for the review in the context of what is already known	p. 3
Objectives	4	Provide an explicit statement of questions being addressed with reference to participants, interventions, comparisons, outcomes, and study design (PICOS)	n/a
*4. Methods*			
Protocol and registration	5	Indicate if a review protocol exists, if and where it can be accessed (e.g., Web address), and, if available, provide registration information including registration number	n/a
Eligibility criteria	6	Specify study characteristics (e.g., PICOS, length of follow-up) and report characteristics (e.g., years considered, language, publication status) used as criteria for eligibility, giving rationale	p. 5
Information sources	7	Describe all information sources (e.g., databases with dates of coverage, contact with study authors to identify additional studies) in the search and date last searched	p. 4 and 8
Search	8	Present full electronic search strategy for at least one database, including any limits used, such that it could be repeated	“Appendix 1”
Study selection	9	State the process for selecting studies (i.e., screening, eligibility, included in systematic review, and, if applicable, included in the meta-analysis)	p. 4
Data collection process	10	Describe method of data extraction from reports (e.g., piloted forms, independently, in duplicate) and any processes for obtaining and confirming data from investigators	p. 4
Data items	11	List and define all variables for which data were sought (e.g., PICOS, funding sources) and any assumptions and simplifications made	n/a
Risk of bias in individual studies	12	Describe methods used for assessing risk of bias of individual studies (including specification of whether this was done at the study or outcome level), and how this information is to be used in any data synthesis	n/a
Summary measures	13	State the principal summary measures (e.g., risk ratio, difference in means)	n/a
Synthesis of results	14	Describe the methods of handling data and combining results of studies, if done, including measures of consistency (e.g., I^2^) for each meta-analysis	n/a
Risk of bias across studies	15	Specify any assessment of risk of bias that may affect the cumulative evidence (e.g., publication bias, selective reporting within studies)	n/a
Additional analyses	16	Describe methods of additional analyses (e.g., sensitivity or subgroup analyses, meta-regression), if done, indicating which were pre-specified	p. 5
*5. Results*			
Study selection	17	Give numbers of studies screened, assessed for eligibility, and included in the review, with reasons for exclusions at each stage, ideally with a flow diagram	p. 8
Study characteristics	18	For each study, present characteristics for which data were extracted (e.g., study size, PICOS, follow-up period) and provide the citations	p. 10
Risk of bias within studies	19	Present data on risk of bias of each study and, if available, any outcome level assessment (see item 12)	n/a
Results of individual studies	20	For all outcomes considered (benefits or harms), present, for each study: (a) simple summary data for each intervention group (b) effect estimates and confidence intervals, ideally with a forest plot	n/a
Synthesis of results	21	Present results of each meta-analysis done, including confidence intervals and measures of consistency	n/a
Risk of bias across studies	22	Present results of any assessment of risk of bias across studies (see Item 15)	n/a
Additional analysis	23	Give results of additional analyses, if done (e.g., sensitivity or subgroup analyses, meta-regression [see Item 16])	p. 12
*6. Discussion*			
Summary of evidence	24	Summarize the main findings including the strength of evidence for each main outcome; consider their relevance to key groups (e.g., healthcare providers, users, and policy makers)	p. 15
Limitations	25	Discuss limitations at study and outcome level (e.g., risk of bias), and at review-level (e.g., incomplete retrieval of identified research, reporting bias)	pp. 14–15
Conclusions	26	Provide a general interpretation of the results in the context of other evidence, and implications for future research	p. 15
*7. Funding*			
Funding	27	Describe sources of funding for the systematic review and other support (e.g., supply of data); role of funders for the systematic review	p. 26

*Based on Moher et al. [20].

## Data Availability

The datasets used and/or analysed during the current study are available from the corresponding author on reasonable request.
